# Risk Factors for Developing Nonmelanoma Skin Cancer after Lung Transplantation

**DOI:** 10.1155/2019/7089482

**Published:** 2019-03-10

**Authors:** Nikolai Gräger, Mareike Leffler, Jens Gottlieb, Jan Fuge, Gregor Warnecke, Ralf Gutzmer, Imke Satzger

**Affiliations:** ^1^Department of Dermatology and Allergy, Skin Cancer Center Hannover, Hannover Medical School, Carl-Neuberg-Str. 1, 30625 Hannover, Germany; ^2^Department of Respiratory Medicine, Hannover Medical School, Carl-Neuberg-Str. 1, 30625 Hannover, Germany; ^3^Department of Cardiac, Thoracic, Transplantation and Vascular Surgery, Hannover Medical School, Carl-Neuberg-Str. 1, 30625 Hannover, Germany

## Abstract

**Background:**

Nonmelanoma skin cancer (NSMC) is the most common malignancy after organ transplantation. Lung transplant recipients (LTRs) are particularly prone to develop NMSC as compared to renal or hepatic transplant recipients due to higher dosages of immunosuppression needed. Everolimus, an immunosuppressant used in organ transplant recipients, is thought to inherit a lower risk for NMSC than calcineurin inhibitors, especially in renal transplant recipients. It is currently unknown whether this also applies to LTRs.

**Objectives:**

To determine risk factors for NMSC and precancerous lesions after lung transplantation (LTx) and to characterize the effect of everolimus-based regimens regarding this risk.

**Materials and Methods:**

90 LTRs and former participants of the interventional trial “Immunosuppressive Therapy with Everolimus after Lung Transplantation”, who were randomized to receive either an everolimus- or mycophenolate mofetil- (MMF-) based regimen, were enrolled and screened in this retrospective, single-center cohort study.

**Results:**

After a median follow-up of 101 months, we observed a prevalence of 38% for NMSC or precancerous lesions. 33% of the patients continuously receiving everolimus from LTx to dermatologic examination compared to 39% of all other patients, predominantly receiving an MMF-based regimen, were diagnosed with at least one NMSC or precancerous lesion (*P=*.66). Independent risk factors for NMSC or precancerous lesions after LTx were male sex and duration of voriconazole therapy.

**Conclusion:**

NMSC or precancerous lesions were very common after LTx, and risk factors were similar to previous reports on LTRs. Everolimus did not decrease this risk under the given circumstances of this study. Patients should be counseled regarding their risk, perform vigorous sunscreen, and undergo regular dermatological controls, regardless of their immunosuppressive regimen.

## 1. Introduction

Since the first successful solid organ transplantation in December 1954 executed by Murray and his associates [1, 2], short-term survival of organ transplant recipients (OTRs) has been improved drastically [3]. New problems challenge physicians arising within the long-term follow-up of OTRs, one of those being posttransplant malignancies. The most common posttransplant malignancy in OTRs is nonmelanoma skin cancer (NMSC), especially cutaneous squamous cell carcinoma (SCC) and basal cell carcinoma (BCC). The risk for SCC and BCC is increased by 65- to 250-fold [4–8] and by 10-fold [9], respectively. Besides, the BCC-to-SCC ratio, which is approximately 4:1 in immunocompetent individuals, is almost completely reversed in OTRs [8], thus indicating the high risk for SCC in OTRs. Not only the risk for NMSC in OTRs is increased but also the aggressiveness in growth and metastasis formation, leading to higher mortality [10, 11].

General risk factors for posttransplant NMSC have been studied well and are described as chronic sun exposure, fair skin, male gender, history of NMSC before transplantation, higher age at transplantation, number, and dosage of immunosuppressants, and type of transplanted organ, especially heart, and lung transplantation [12–17]. Investigations focusing on lung transplant recipients (LTRs) found similar risk factors for posttransplant NMSC, namely higher age at lung transplantation (LTx), high sun exposure, fair skin, male sex, history of skin cancer before LTx and duration and dosage of voriconazole intake for prophylaxis or therapy of invasive fungal infections [18–27]. Data from clinical studies regarding primary and secondary prevention of NMSC in renal transplant recipients (RTRs) suggest a lower risk for NMSC development from mammalian target of rapamycin inhibitors (mTORis), such as everolimus or sirolimus, in contrast to calcineurin inhibitor- (CNI-) based immunosuppressive regimens [28–33]. However, studies on the role of the immunosuppressive regimen in LTRs are missing [23].

In addition to NMSC, its precancerous lesions, such as actinic keratoses (AKs), actinic cheilitis (AC), and Bowen's disease (BD), are also often found in OTRs, especially on sun-exposed skin [34]. Those entities tend to transform into SCC [35–37], although there still is a lack of consistent data regarding conversion rates [38]. Nevertheless, the number of precancerous lesions is a strong indicator of the risk for developing NMSC in OTRs [39]. Following those findings, it is necessary to identify and treat not only NMSC but also its precancerous lesions.

This retrospective, single-center cohort study performed detailed dermatologic history and examinations in LTRs who participated in a clinical study. Within this study, patients were randomized in two groups with different immunosuppressive regimens, either with everolimus or with mycophenolate mofetil (MMF) [40]. Thus, our study aimed to identify risk factors for NMSC and its precancerous lesions after LTx and to characterize the effect of an mTORi-based regimen on this risk.

## 2. Materials and Methods

### 2.1. Patients

This retrospective, single-center cohort study was approved by the Ethics Committee of Hannover Medical School on March 23, 2015 (approval no. 2646-2015), and conducted in accordance with the Helsinki Declaration of 1975, as revised in 1983. All participants provided written informed consent.

Between July 2015 and January 2016, we screened LTRs for NMSC or precancerous lesions at our Skin Cancer Center at Hannover Medical School, Germany. Patients were in primary treatment within the LTx program of the Department of Cardiac, Thoracic, Transplantation and Vascular Surgery and aftercare via the LTx outpatient clinic of the Department of Respiratory Medicine, both at Hannover Medical School.

All patients completed the open-label, prospective, randomized, single-center trial “Immunosuppressive therapy with Certican® (Everolimus) after lung transplantation”, ClinicalTrials.gov ID: NCT00402532. This trial randomized patients undergoing LTx in the years 2005–2009 into two study arms. The control group received the standard triple-immunosuppression with MMF, prednisolone, and high dose CsA. The everolimus group received the comparative triple-immunosuppression with everolimus, prednisolone, and low dose CsA. The study included a follow-up of two years after LTx [40]. The interval between LTx and inclusion in our dermatological study was five years at least.

### 2.2. Dermatologic Examination

Medical histories of all participants were taken via a structured dermatologic questionnaire for common skin diseases, especially NMSC and precancerous lesions, exposure to UV radiation, habits of using sunscreen products, and history of malignancies before and after LTx. Special attention was paid to previous dermatologic diseases or skin biopsies and surgeries. A general dermatologic exam assessing Fitzpatrick skin type, signs of chronic sun damage, and the number of typical and atypical melanocytic naevi was performed. Furthermore, patients were screened with dermoscopy and optical coherence tomography for any skin cancer or precancerous lesion by a board-certified dermatologist. All observers were blinded regarding the immunosuppressive regimen at the date of dermatologic examination. For each patient, the day-exact history of immunosuppressive regimen and voriconazole therapy was evaluated.

### 2.3. Statistical Analysis

Data was stored in a database using FileMaker Pro for Windows, version twelve (FileMaker, Inc., Santa Clara, CA, USA), and double-checked against original records before analyses. Medication records were stored using Microsoft Excel 2010, version 14 (Microsoft Corporation, Redmond, WA, USA). Statistical analyses were performed using IBM SPSS Statistics for Windows, version 23 (IBM Corp., Armonk, NY, USA). Data on patient baseline parameters were described as medians with range and proportions with percentages as suitable. Qualitative variables were analyzed using *χ*^2^-Test or Fisher exact test if required. Quantitative variables without a normal distribution were analyzed using Mann-Whitney-U test. Normal distribution was tested using Shapiro-Wilk and Kolmogorov-Smirnov-Test. Multiple testing was addressed using Bonferroni correction. For multivariate analysis, we used binary logistic regression with a backward conditional approach including variables with a* P* value <.2 in univariate analysis. Also, we used Cox regression with a backward conditional approach and an exclusion threshold of a* P* value ≤.1. The assumption of proportional hazards was tested with complementary log-log plots for dichotomous variables. We used Kaplan-Meier method with log-rank test calculating tumor-free survival. Results of the “further findings” section were calculated as post hoc analyses and without correction for multiplicity. All tests were two-sided. A* P* value <.05 was considered statistically significant in all statistical methods.

## 3. Results

### 3.1. Study Population

The previous interventional trial “Immunosuppressive Therapy with Everolimus after Lung Transplantation”, carried out between 2005 and 2011, comprised 190 participants. After first LTx and randomization to receive either an MMF- or everolimus-based immunosuppressive regimen, 97/190 (51.1%) patients completed the two years on the study drug. Discontinuation of everolimus occurred in 52/95 (55%) patients and of MMF in 41/95 (43%) patients. The most common reasons for discontinuation were recurrent acute rejection or onset of bronchiolitis obliterans syndrome. After discontinuation, alternative immunosuppressants, such as tacrolimus, azathioprine, or sirolimus, were administered. In addition to immunosuppressive therapy, all patients received either itraconazole or voriconazole preventing mycotic infection.

**Figure 1:**
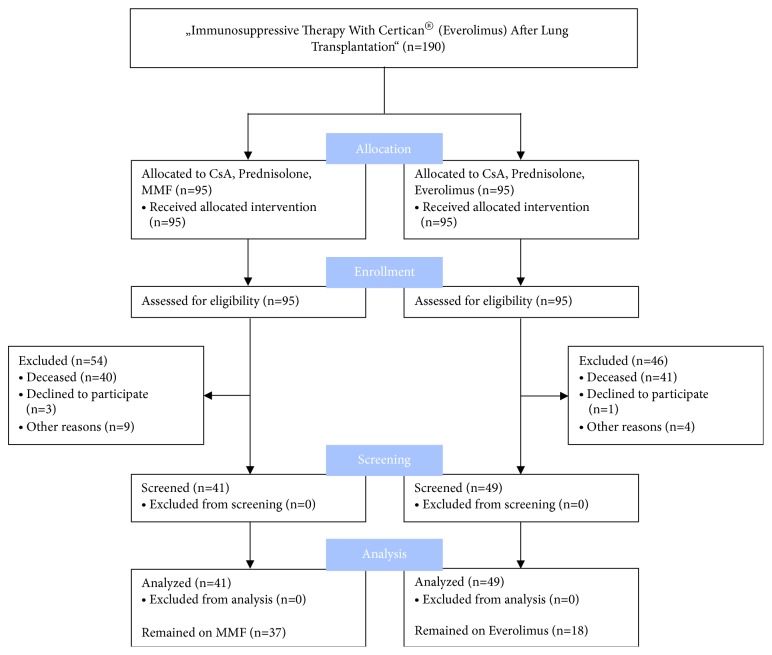
Adapted CONSORT 2010 Flow Diagram. Distribution of all potential and definite participants at each stage of the study. CsA, cyclosporine A; MMF, mycophenolate mofetil.

In this study, it was possible to include 90 participants of the initial trial, 49/95 (52%) from the former everolimus arm, and 41/95 (43%) from the former MMF arm, referred to as “quasi intention to treat”. 18/95 (19%) patients from the former everolimus arm continuously received everolimus until dermatologic examination, referred to as “quasi per protocol”. 37/95 (39%) patients from the former MMF arm received MMF until dermatologic examination. No missing data were identified; all participants were included in the statistical analyses (Figure 1).

Patient demographic characteristics are displayed in Table 1. Regarding age, sex, follow-up period, Fitzpatrick skin type, hair color, history of pretransplant cancer, underlying disease, transplant type, and voriconazole exposure there were no significant differences between the evaluated groups. The nine different immunosuppressive regimens administered at dermatologic exam are shown in Table 2.

### 3.2. Skin Cancer Prevalence

After a median follow-up of 101 (range 69–128) months from first LTx to date of dermatologic examination, 34 (38%) patients were diagnosed with NMSC or precancerous lesions, 32 (36%) patients with precancerous lesions, 16 (18%) patients with NMSC, 14 (16%) patients with NMSC and precancerous lesions and three (3%) patients with other malignant or semimalignant entities of the skin (lentiginous melanoma in situ, melanoma in situ, cornu cutaneum). In the precancerous lesions' subgroup, 18 patients (20%) had AKs, twelve patients (13%) had BD, and seven patients (7%) had AC. In the NMSC subgroup, ten (11%) patients developed SCC, one of them (1%) metastasized, and nine patients (10%) developed BCC. One patient (1%) developed a posttransplant malignancy other than skin cancer (pancreatic carcinoma).

### 3.3. Skin Cancer Depending on Immunosuppression

The posttransplant skin cancer prevalences for patients from the quasi per protocol group compared to all other participants at the date of dermatologic examination were as follows. NMSC or its precancerous lesions were found in 33% of the everolimus arm and 39% in the comparator arm (*P*=.66). Precancerous lesions were found in 28% versus 38% (*P*=.44). AKs were found in 17% versus 21% (*P*>.99), BD was found in 6% versus 15% (*P*=.45), and AC was found in 6% versus 8% (*P*>*.99*). NMSC was found in 6% versus 21% (*P*=.18), SCC was found in 0% versus 14% (*P*=.20), BCC was found in 6% versus 11% (*P*=.68). Other malignant or semimalignant entities were found in 0% versus 4% (*P*>.99), being acral lentiginous melanoma in situ, melanoma in situ, and cornu cutaneum (Table 3).

**Figure 2:**
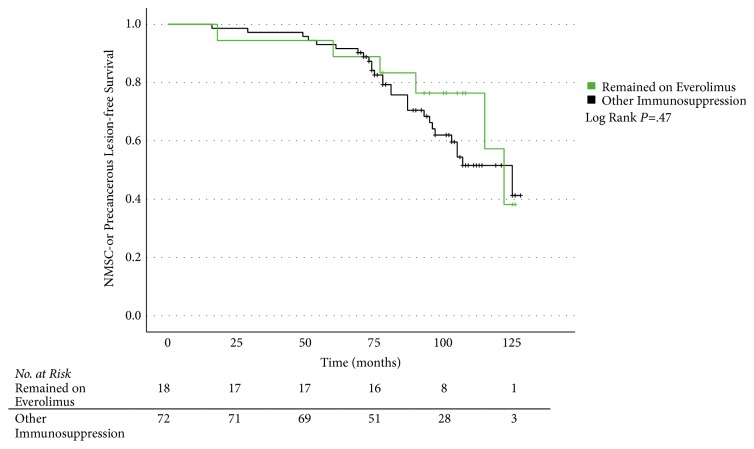
Skin cancer-free survival after lung transplantation. Kaplan-Meier plot showing the NMSC- or precancerous lesion-free survival after first lung transplantation comparing patients from the former everolimus arm, who remained on everolimus until dermatologic exam (“quasi per protocol”), to all other patients. Overall, 90 patients are included and analyzed with log-rank test for difference in median skin cancer-free survival (*P=*.47). NMSC, nonmelanoma skin cancer.

Besides the prevalence rates, we analyzed the NMSC- or precancerous lesion-free survival between the groups mentioned above with a Kaplan-Meier plot, showing a median tumor-free survival of 122 months (95% CI 107.8–136.2) versus 125 months (95% CI 99.5–150.5) (*P*=.47) (Figure 2).

### 3.4. Risk Factors for Skin Cancer

Finally, risk factors for NMSC or its precancerous lesions were calculated, using binary logistic regression (Table 4). Variables tested for inclusion in multivariate analysis via univariate analyses were male sex, age at LTx, fair skin, duration of voriconazole intake, long-term immunosuppression without everolimus, high UV exposure before and after LTx, and minimal usage of sunscreen products before and after LTx. Male sex, age at first LTx, fair skin, duration of voriconazole intake and high UV exposure after LTx revealed a* P* value <.2 in univariate analysis and were therefore included in multivariate analysis. Male sex (OR 4.01, 95% CI 1.43–11.22,* P=*.008), higher age at first LTx (OR 1.06, 95% CI 1.01–1.12,* P=*.02), fair skin (OR 3.01, 95% CI 1.02–8.93,* P=*.047), and duration of voriconazole intake (OR 1.11, 95% CI 1.00–1.23,* P=*.04) appeared to be independent risk factors for NMSC or its precancerous lesions after LTx.

Accounting for differences in the follow-up times of the participants, we also calculated a Cox proportional-hazards model. Variables tested for inclusion in the final model were male sex, age at LTx, fair skin, duration of voriconazole exposure, long-term immunosuppression without everolimus, high UV exposure before and after LTx, and minimal usage of sunscreen products before and after LTx. The final Cox proportional-hazards model revealed male sex (HR 2.71, 95% CI 1.24–5.94,* P*=.01) and duration of voriconazole exposure (HR 1.03, 95% CI 1.00–1.06,* P*=.04) as risk factors for NMSC or its precancerous lesions after LTx after adjusting for each other (Table 5).

### 3.5. Further Findings

Univariate subgroup analyses revealed additional results. The intake of voriconazole longer than six months was associated with a higher risk for AC. The risk was increased by almost 9-fold (RR 8.67, 95% CI 2.21–34.04,* P=*.005). The risk for posttransplant NMSC in patients with a posttransplant diagnosis of precancerous lesions was increased by 13-fold (RR 12.69, 95% CI 3.08–52.35,* P<*.001). LTRs, who stated to pursue outdoor activities regularly after transplantation had an increased risk for AK by 3-fold (RR 2.55, 95% CI 1.15–5.63,* P=*.044).

## 4. Discussion

In this single-center, retrospective cohort study, we aimed to identify risk factors for NMSC and its precancerous lesions in LTRs and to investigate the influence of everolimus-based regimens on this risk. Within 90 LTRs, we detected a prevalence for NMSC or precancerous lesions of 38% and a prevalence for NMSC of 18% after a median follow-up of 101 months. This prevalence was noticeably higher than in a large population-based study by Krynitz et al. [7] of 10,476 mixed OTRs (kidney, liver, heart, lung, pancreas, and small intestine) with a median follow-up ranging from four years (pancreas or small intestine) to eight years (kidney). They found an SCC prevalence of 6.4% (668/10,476). In the subgroup of heart and/or lung transplant recipients with a median follow-up of five (0–23) years, an SCC prevalence of 5.9% (60/1,012) was found. Precancerous lesions and BCC were not evaluated.

Analogous to the work of Feist et al. with LTRs [20] and Ducroux et al. with liver transplant recipients [41], we firstly used binary logistic regression to identify risk factors for NMSC or precancerous lesions after LTx. We found that male sex, higher age at transplantation, Fitzpatrick skin types I and II and duration of exposure to voriconazole were independent risk factors. Feist et al. found that higher age at the time of transplant, history of skin cancer pretransplant, and extended voriconazole therapy were risk factors for NMSC after LTx [20]. Those findings were similar to ours, except for history of pretransplant skin cancer. This is to the fact that just one of our patients had a history of pretransplant skin cancer and we, therefore, did not include this variable in our models.

However, binary logistic regression is not a robust method to use when dealing with different follow-up periods [42], which applied to our population. Therefore, those results have to be interpreted with caution. In consequence, we additionally followed a time-to-event approach by calculating a Cox proportional-hazards model. Here we also found that male sex and duration of voriconazole intake were risk factors for NMSC or its precancerous lesions after LTx. Higher age at transplantation and fair skin types were not significant risk factors but still exhibited a trend regarding an increased risk for NMSC or its precancerous lesions.

A recently published large multicenter cohort study of 10,649 mixed OTRs also found that male sex, higher age at transplantation, white race, and transplantation of thoracic organs are risk factors for posttransplant skin cancer [43]. A retrospective study of an Israeli population of LTRs describing malignancies after transplantation reported a prevalence of 15.7% (16/102) for any malignancy, the most common cancer was NMSC with 9.8% (10/102). The patients diagnosed with cancer were significantly older, mostly male, and also mostly past smokers [18]. In our study, we did not evaluate the current or former smoking status.

A retrospective study focusing on SCC and voriconazole exposure after LTx with 543 patients found a prevalence of 3.1% (17/543) for SCC after transplantation. The patients who developed SCC were analyzed with a 1:3 case-control approach. In multivariate analysis, they identified high levels of sun exposure and duration of voriconazole therapy as risk factors for SCC after LTx [19]. The rather low number of skin cancers was most likely due to the short median follow-up of 36 months. Also, a drawback of this study was a significant difference between the cases and controls within the demographics regarding age, gender, and residence in regions with high sun exposure.

Further studies on LTRs also described male sex, older age at transplantation, history of pretransplant skin cancer, Fitzpatrick skin types I and II, and dose and duration of voriconazole therapy as risk factors for posttransplant NMSC [20–22, 25].

McLaughlin et al. on the contrary did not find voriconazole as a risk factor for NMSC after LTx but male sex, higher age, sun exposure, history of chronic obstructive pulmonary disorder, and history of immune disorder. They discussed, that most of the results regarding voriconazole from previous studies were not controlled for confounders, such as patient gender, history of COPD, and history of immune disorder, and therefore not conclusive [44].

In contrast to this and support to our findings, more recent and more extensive studies with 400 [25], 455 [24], and 900 [26] LTRs demonstrated voriconazole intake as a risk factor for NMSC after LTx. Responsible for this effect presumably is the metabolite voriconazole N-oxide since it promotes phototoxicity [27].

Our findings regarding long-term immunosuppression with everolimus in combination with CNI in comparison to MMF show no significantly decreased risk for NMSC or its precancerous lesions after LTx. Nevertheless, there was a trend for a decreased prevalence of NMSC in the everolimus-treated group. This may have occurred due to the lack of statistical power of the quasi per protocol group with its small cohort size of 18 patients. A reason for the small cohort might be the high rate of discontinuation of mTORis. This has been demonstrated before in other reports because of adverse events such as pneumonitis, edema, proteinuria, diarrhea, dyslipidemia, anemia, acne-like lesions, aphthous ulcers, and other side effects [45, 46]. Also, the overall number of participants in our study was determined through the size of the previous interventional trial and particularly limited to the survival of these patients, as 42% (40/95) from the MMF arm and 43% (41/95) from the everolimus arm deceased before inclusion in our study.

To our knowledge, our study is the second to investigate the effect of mTORis in LTRs. Rashtak et al. [23] evaluated 166 LTRs in a single-center, retrospective cohort study regarding the incidence and risk factors of skin cancer. After a median follow-up of three (range 0–21) years, 47/166 (28.3%) LTRs were diagnosed with 162 SCCs, 45 BCCs, one malignant melanoma, one Merkel cell carcinoma, and one atypical fibroxanthoma, precancerous lesions were not evaluated. Forty-four patients (26.5%) developed at least one SCC and 19 patients (11.4%) at least one BCC. Our findings regarding SCC (11%) are lower than those mentioned above despite the shorter follow-up period of three years as compared to eight years in our cohort. On the contrary, our findings regarding BCC (10%) are similar to those of Rashtak and colleagues. Congruent to our findings, they also found that an mTORi-based immunosuppressive regimen with sirolimus did not decrease the risk, while increased age, male sex, history of skin cancer, and more recent year of transplantation were associated with a higher risk. Those results partially overlap with our findings, even though our patients received everolimus instead of sirolimus.

In the literature, we found no randomized, controlled trial with LTRs regarding the effect of mTORis on posttransplant NMSC. The current prospective, randomized trials evaluating this effect were all performed with RTRs. Four out of five studies managed to show a reduced risk for NMSC within the mTORi arm [32, 33, 45, 47, 48].

A single retrospective study with a large cohort of mixed OTRs by Karia et al. [29] showed a decreased risk for subsequent skin cancer after developing posttransplant cancer of any type by switching immunosuppression to sirolimus. Although the cohort consisted of mixed OTRs, more than half of the patients were RTRs. In the nonrenal transplant subgroup, the risk reduction for secondary skin cancer appeared to be insignificant.

Most of these studies mentioned above showed a decreased risk for NMSC after renal transplantation by switching immunosuppression from a CNI to an mTORi. A recently published review on posttransplant skin cancer summarized that the most beneficial effect of mTORis to reduce posttransplant NMSC occurs through the early conversion from CNIs to mTORis and the reduction of CNI dose by simultaneously introducing an mTORi [49]. In our cohort, most patients remained on a CNI and received an mTORi instead of MMF or azathioprine. As CNIs are a known risk factor for posttransplant NMSC [50–52], this may be a reason, why we did not find a reduced risk.

Our patients received a triple immunosuppressive regimen, like most RTRs [53]. On the contrary, the dosage needed to prevent organ rejection is higher in LTRs, than in RTRs [29]. Higher dosage and triple immunosuppressive therapy are known to increase the risk for posttransplant NMSC [54]. This as well might be a reason for the similar prevalences of NMSC or its precancerous lesions of the observed groups. Finally, our median follow-up period of 101 months was considerably longer than those of the prospective trials with one year [32], 1.68, and 1.74 years (mean) for the sirolimus and the CNI group [48] and two years [33, 45, 47], giving us the opportunity to diagnose NMSC, that develop later after transplantation.

Besides the strengths of our study which are the long follow-up period and the day-exact analyses of the immunosuppressive regimens, it also has some limitations. The retrospective design, the single-center setting, recall bias due to the assessment of medical history via a questionnaire and the rather small cohort size, especially the limited number of patients with long-term everolimus usage are disadvantages. Moreover, we did not include and investigate the deceased patients from the previous interventional trial. Although the medication history of the immunosuppressive regimens was provided day-exact, the patients' adherence to medication intake could not be assessed but should not differ between the observed groups.

## 5. Conclusion

Our study showed a high prevalence of NMSC and its precancerous lesions of 38% in LTRs. We confirmed some of the known risk factors for posttransplant malignancies of the skin. Men who received voriconazole therapy were particularly prone to develop NMSC and its precancerous lesions. In this small cohort, it appeared that long-term immunosuppression with an everolimus-based regimen did not decrease the risk of skin cancer after LTx as compared to MMF-based regimens. The high risk for NMSC after LTx and the lack of consistent data concerning those patients emphasize the need for prospective trials regarding LTRs and the effect of mTORis on the risk for posttransplant skin cancer. Until then, it is necessary to be aware of the risk factors, to educate LTRs regarding those risk factors, and to observe those with high-risk profile closely for malignancies of the skin, regardless of the immunosuppressive regimen.

## Figures and Tables

**Table 1:** Patient demographic characteristics.

		Quasi Intention to Treat^a^			Quasi Per Protocol^b^		
	All Patients	Everolimus Arm	MMF Arm		Remained on Everolimus	Other Immunosuppression	
Characteristic^c^	(n=90)	(n=49)	(n=41)	*P* Value	(n=18)	(n=72)	*P* Value
Age, median (range), y	56 (28 – 71)	53 (28 – 67)	58 (29 – 71)	.06^d^	58 (31 – 67)	53 (28 – 71)	.27^d^
Sex, No. (%)							
Female	41 (46)	24 (49)	17 (42)	.48^e^	11 (61)	30 (42)	.14^e^
Male	49 (54)	25 (51)	24 (59)		7 (39)	42 (58)	
Follow-up, median (range), m	101 (69 – 128)	101 (69 – 126)	102 (73 – 128)	.55^d^	103 (77 – 126)	101 (69 – 128)	.35^d^
Fitzpatrick skin type, No. (%)							
I	13 (14)	6 (12)	7 (17)	.21^f^	1 (6)	12 (17)	.48^f^
II	46 (51)	29 (59)	17 (42)		12 (67)	34 (47)	
III	29 (32)	14 (29)	15 (37)		5 (28)	24 (33)	
IV	2 (2)	0 (0)	2 (5)		0 (0)	2 (3)	
Hair color, No. (%)							
Red	7 (8)	3 (6)	4 (10)	.56^f^	1 (6)	6 (8)	.62^f^
Light blonde	11 (12)	8 (16)	3 (7)		3 (17)	8 (11)	
Dark blonde	46 (51)	25 (51)	21 (51)		7 (39)	39 (54)	
Brown	25 (28)	13 (27)	12 (29)		7 (39)	18 (25)	
Black	1 (1)	0 (0)	1 (2)		0 (0)	1 (1)	
History of pre-transplant cancer, No. (%)							
Skin							
Yes	1 (1)	0 (0)	1 (2)	.46^f^	0 (0)	1 (1)	>.99^f^
No	89 (99)	49 (100)	40 (98)		18 (100)	71 (99)	
Other							
Yes	2 (2)	1 (2)	1 (2)	>.99^f^	0 (0)	2 (3)	>.99^f^
No	88 (98)	48 (98)	40 (98)		18 (100)	70 (97)	
Underlying disease, No. (%)							
Cystic fibrosis	26 (29)	14 (29)	12 (29)	.23^f^	2 (11)	24 (33)	.11^f^
Emphysema	32 (36)	14 (29)	18 (44)		6 (33)	26 (36)	
Pulmonary fibrosis	19 (21)	14 (29)	5 (12)		7 (39)	12 (17)	
Other	13 (14)	7 (14)	6 (15)		3 (17)	10 (14)	
Transplant type, No. (%)							
Bilateral	84 (93)	45 (92)	39 (95)	.69^f^	16 (89)	68 (94)	.60^f^
Unilateral	6 (7)	4 (8)	2 (5)		2 (11)	4 (6)	
Voriconazole exposure, No. (%)							
Yes	40 (44)	22 (45)	18 (44)	.93^e^	5 (28)	35 (49)	.11^e^
No	50 (56)	27 (55)	23 (56)		13 (72)	37 (51)	
>6 months^g^	12 (13)	7 (14)	5 (12)	.77^e^	2 (11)	10 (14)	>.99^f^
≤6 months^g^	78 (87)	42 (86)	36 (88)		16 (89)	62 (86)	

MMF, mycophenolate mofetil.

^a^Patients stratified by original therapy arms from the previous interventional trial “Immunosuppressive therapy with Certican® (Everolimus) after lung transplantation”.

^b^Comparing patients from the former everolimus arm, who remained on everolimus until dermatologic exam to all other patients.

^c^Percentages have been rounded to whole numbers and may not add up to 100.

^d^Calculated using the Mann-Whitney-U test.

^e^Calculated using the *χ*^2^-test.

^f^Calculated using the Fisher exact test.

^g^Calculated cumulating all periods of voriconazole intake.

**Table 2:** Immunosuppressive regimens at dermatologic examination.

		Quasi Intention to Treat^a^		Quasi Per Protocol^b^	
	All Patients	Everolimus Arm	MMF Arm	Remained on Everolimus	Other immunosuppression
Immunosuppressive Regimen, No. (%)^c^	(n=90)	(n=49)	(n=41)	(n=18)	(n=72)
Tacrolimus, MMF, prednisolone	35 (39)	16 (33)	19 (46)	0 (0)	35 (49)
CsA, MMF, prednisolone	29 (32)	10 (20)	19 (46)	0 (0)	29 (40)
CsA, everolimus, prednisolone	16 (18)	16 (33)	0 (0)	15 (83)	1 (1)
Tacrolimus, azathioprine, prednisolone	3 (3)	2 (4)	1 (2)	0 (0)	3 (4)
Tacrolimus, everolimus, prednisolone	3 (3)	2 (4)	1 (2)	2 (11)	1 (1)
CsA, everolimus, MMF, prednisolone	1 (1)	1 (2)	0 (0)	1 (6)	0 (0)
MMF, sirolimus, prednisolone	1 (1)	1 (2)	0 (0)	0 (0)	1 (1)
Tacrolimus, MMF	1 (1)	0 (0)	1 (2)	0 (0)	1 (1)
Tacrolimus, prednisolone	1 (1)	1 (2)	0 (0)	0 (0)	1 (1)

MMF, mycophenolate mofetil; CsA, cyclosporine A.

^a^Patients stratified by original therapy arms from the previous interventional trial “Immunosuppressive therapy with Certican® (Everolimus) after lung transplantation”.

^b^Comparing patients from the former everolimus arm, who remained on everolimus until dermatologic exam to all other patients.

^c^Percentages have been rounded to whole numbers and may not add up to 100.

**Table 3:** Posttransplant skin cancer by treatment groups^a^.

		Quasi Intention to Treat^b^			Quasi Per Protocol^c^		
	All Patients	Everolimus Arm	MMF Arm		Remained on Everolimus	Other Immunosuppression	
Skin Cancer, No. (%)^d^	(n=90)	(n=49)	(n=41)	*P* Value^e^	(n=18)	(n=72)	*P* Value^e^
Precancerous lesions or NMSC	34 (38)	18 (37)	16 (39)	.82^f^	6 (33)	28 (39)	.66^f^
Precancerous lesions	32 (36)	16 (33)	16 (39)	.53^f^	5 (28)	27 (38)	.44^f^
AK	18 (20)	9 (18)	9 (22)	.67^f^	3 (17)	15 (21)	>.99
BD	12 (13)	8 (16)	4 (10)	.36^f^	1 (6)	11 (15)	.45
AC	7 (8)	2 (4)	5 (12)	.24	1 (6)	6 (8)	>.99
NMSC	16 (18)	9 (18)	7 (17)	.87^f^	1 (6)	15 (21)	.18
SCC	10 (11)	5 (10)	5 (12)	>.99	0 (0)	10 (14)	.20
BCC	9 (10)	6 (12)	3 (7)	.50	1 (6)	8 (11)	.68
Other^g^	3 (3)	2 (4)	1 (2)	>.99	0 (0)	3 (4)	>.99

MMF, mycophenolate mofetil; NMSC, nonmelanoma skin cancer; AK, actinic keratosis; BD, Bowen's disease; AC, actinic cheilitis; SCC, squamous cell carcinoma of the skin; BCC, basal cell carcinoma.

^a^Two superordinate groups, each with nine tests, Bonferroni correction (0.05/9 = 0.0056): *P* values < .0056 are deemed to be significant.

^b^Patients stratified by original therapy arms from the previous interventional trial “Immunosuppressive therapy with Certican® (Everolimus) after lung transplantation”.

^c^Comparing patients from the former everolimus arm, who remained on everolimus until dermatologic exam, to all other patients.

^d^Percentages have been rounded to whole numbers and may not add up to 100.

^e^Unless otherwise indicated, calculated using the Fisher exact test.

^f^Calculated using the *χ*^2^-test.

^g^Other entities found were acral lentiginous melanoma in situ (n=1), melanoma in situ (n=1), and cornu cutaneum (n=1).

**Table 4:** Binary logistic regression of risk factors for NMSC or precancerous lesions after lung transplantation.

	OR (95% CI)			
Variable	Univariate Analysis	*P* value	Multivariable Analysis^a^	*P* value
Male sex	2.41 (0.99 – 5.88)	.05	4.01 (1.43 – 11.22)	.008
Higher age at first lung transplantation, y	1.04 (0.99 – 1.09)	.06	1.06 (1.01 – 1.12)	.02
Fair skin^b^	2.27 (0.87 – 5.88)	.09	3.01 (1.02 – 8.93)	.05^c^
Time of voriconazole exposure, m^d^	1.06 (0.99 – 1.14)	.11	1.11 (1.00 – 1.23)	.04
High UV exposure after LTx	0.60 (0.30 – 1.20)	.15	0.76 (0.33 – 1.72)	.50
Minimal sunscreen usage before LTx	1.10 (0.44 – 2.75)	.84	NA	NA
High UV exposure before LTx	0.86 (0.45 – 1.66)	.65	NA	NA
Minimal sunscreen usage after LTX	2.19 (0.61 – 7.81)	.23	NA	NA
Immunosuppression without everolimus^e^	1.27 (0.43 – 3.78)	.66	NA	NA

OR, odds ratio; CI, confidence interval; UV, ultra violet; NA, not applicable.

^a^The final model exhibited an overall significance (*P=*.001, Nagelkerkes *R*^*2*^=.27).

^b^Representing Fitzpatrick skin types I and II.

^c^*P*=.047.

^d^Calculated cumulating all periods of voriconazole intake.

^e^Patients not continuously receiving everolimus from LTx to dermatologic examination.

**Table 5:** Cox regression of risk factors for NMSC or precancerous lesions after lung transplantation.

	HR (95% CI)			
Variable	Initial Model	*P* value	Final Model	*P* value
Male sex	2.69 (1.19 – 6.10)	.02	2.71 (1.24 – 5.94)	.01
Higher age at first lung transplantation, y	1.03 (0.995 – 1.08)	.09	1.03 (0.994 – 1.07)	.10
Fair skin^a^	1.92 (0.69 – 5.26)	.21	2.25 (0.96 – 5.29)	.06
Time of voriconazole exposure, m^b^	1.04 (0.9995 – 1.07)	.053	1.03 (1.00 – 1.06)	.04
High UV exposure before LTx	0.998 (0.24 – 4.16)	.998	NA	NA
High UV exposure after LTx	1.01 (0.19 – 5.43)	.99	NA	NA
Minimal sunscreen usage before LTx	0.73 (0.25 – 2.16)	.57	NA	NA
Minimal sunscreen usage after LTX	2.12 (0.61 – 7.33)	.24	NA	NA
Immunosuppression without everolimus^c^	0.93 (0.37 – 2.39)	.89	NA	NA

HR, hazard ratio; CI, confidence interval; UV, ultra violet; LTx, lung transplantation; NA, not applicable.

^a^Representing Fitzpatrick skin types I and II.

^b^Calculated cumulating all periods of voriconazole intake.

^c^Patients not continuously receiving everolimus from LTx to dermatologic examination.

## Data Availability

The datasets generated and analyzed during the current study are available from the corresponding author on reasonable request.
